# Physiological Responses and Safety Evaluation of Combined Fremont™ Snare and Medetomidine–Ketamine–Acepromazine Immobilization in Free-Ranging Apennine Wolves (*Canis lupus italicus*)

**DOI:** 10.3390/ani16111735

**Published:** 2026-06-04

**Authors:** Simone Angelucci, Fabrizia Di Tana, Catarina Oliveira, José M. Almeida, Marco Carafa, Marta Gandolfi, Lorenzo Petrizzelli, Giovanna Di Domenico, Cristina E. Di Francesco, Camilla Smoglica, Antonio Antonucci

**Affiliations:** 1 Maiella National Park, Wildlife Research Center, Via del Vivaio Snc, 65023 Caramanico Terme, PE, Italyantonio.antonucci@parcomajella.it (A.A.); 2 CECAV—Centro de Ciência Animal e Veterinária, Universidade de Trás-os-Montes e Alto Douro (UTAD), 5000-801 Vila Real, Portugal; 3 Department of Veterinary Medicine, University of Teramo, Loc. Piano D’Accio, 64100 Teramo, TE, Italy

**Keywords:** *Canis lupus italicus*, wildlife capture, chemical immobilization, physiological monitoring, hematology, venous blood gas, capture safety, GPS telemetry, conservation management, Apennine wolf

## Abstract

The Apennine wolf (*Canis lupus italicus*) is a distinct subspecies whose ongoing population recovery in Italy has progressively increased the demand for live capture protocols validated for scientific monitoring and conservation management. Despite the widespread use of mechanical and chemical immobilization in European wolf management, no study has to date systematically evaluated the combined use of the Fremont™ humane foot snare with a medetomidine-ketamine-acepromazine (MKA) protocol in this subspecies, nor characterized the associated cardiorespiratory, thermal, and hematobiochemical parameters under operational field conditions. Between June 2010 and July 2017, thirteen free-ranging Apennine wolves were captured in Maiella National Park (central Apennines, Italy) using the Fremont™ snare and immobilized with a standardized MKA protocol; only animals immobilized with this protocol are reported here, as three additional capture events employed different drug combinations. Cardiorespiratory parameters, body temperature, peripheral oxygen saturation, venous blood gas values, and a comprehensive hematological and serum biochemical panel were recorded during immobilization. Mean heart rate was 100 ± 15 bpm, respiratory rate 24 ± 13 breaths/min, body temperature 38.1 ± 1.3 °C, and mean SpO_2_ 88 ± 11% (range: 66–97%; *n* = 12). No clinically significant hyperthermia requiring active intervention was recorded in the cohort as a whole. Hematological and biochemical values were broadly consistent with published reference ranges for the species, with condition-specific deviations identified in two individuals—one pregnant female and one juvenile presenting signs of transient capture-related myopathy—both of which resolved without clinical sequelae. No capture-related mortality occurred. All thirteen individuals survived the minimum post-capture monitoring period. Preliminary GPS observations in a subset of individuals (*n* = 3) suggest a transient reduction in movement activity in the immediate post-release period. These findings support the safety and operational feasibility of the combined Fremont™ snare–MKA protocol for the Apennine wolf, and provide baseline physiological and hematobiochemical reference data for *Canis lupus italicus* relevant to future capture and conservation management programmes.

## Simple Summary

The Apennine wolf is a genetically unique subspecies that, after nearly disappearing in Italy in the 1970s, has recovered remarkably thanks to decades of legal protection and conservation efforts. Today, scientific monitoring of free-ranging wolves—including live capture, fitting of satellite tracking collars, and biological sampling—is essential both to understand wolf ecology and to manage the increasingly complex relationship between wolves and human activities. However, live capture carries inherent risks, and validated, species-specific protocols for the Apennine wolf have never been formally documented. This study reports on the capture of thirteen free-ranging Apennine wolves in Maiella National Park (central Italy) using a humane foot snare combined with a reversible drug combination (a sedative, a dissociative anaesthetic, and a tranquilizer). We measured heart rate, breathing rate, body temperature, blood oxygen levels, and a comprehensive panel of blood parameters to evaluate the physiological responses of the animals throughout the procedure. All thirteen wolves survived capture with no serious injuries or lasting health effects, and preliminary observations in three individuals suggest a transient reduction in movement activity shortly after release. These results indicate that the protocol described is safe and operationally feasible for this subspecies, and provide a physiological and hematobiochemical reference dataset for the Apennine wolf that may inform future capture and conservation management programmes in Italy.

## 1. Introduction

The recovery of the gray wolf (*Canis lupus*) across Europe represents one of the most well-documented large carnivore conservation successes of recent decades, driven by a combination of legal protection, land-use changes, and recovering prey populations [1,2,3]. Recent continental assessments confirm that this recovery is ongoing: by 2022, at least 21,500 wolves inhabited Europe—an increase of approximately 58% over the preceding decade—with populations expanding in range and number across the great majority of monitored countries [3,4]. This trajectory represents a remarkable conservation achievement for a large predator persisting in heavily human-modified landscapes, and underscores the extraordinary ecological adaptability of the species. In Italy, the Apennine wolf (Canis lupus italicus) underwent severe population decline during the nineteenth and twentieth centuries, reaching a critical low of an estimated 100 individuals in the 1970s [5], as the outcome of a deliberate and sustained effort to remove a perceived threat to livestock breeding and, occasionally, to human safety. Subsequent recovery was facilitated by the enactment of national protective legislation and later reinforced by the European Union Habitats Directive, which afforded the species strict legal protection across its range. A key ecological driver of this demographic rebound was the widespread post-World War II abandonment of rural mountain and hill areas, which created available territories for recolonization and coincided with the resurgence of wild ungulate populations—including wild boar (Sus scrofa), roe deer (Capreolus capreolus), and red deer (Cervus elaphus)—thereby restoring the trophic conditions necessary to sustain expanding wolf populations [6,7,8,9,10]. The Italian population currently numbers at least 3000 individuals and continues to expand its range northward into the Alps and beyond [11,12]. Beyond its conservation status, the Apennine wolf represents a case of remarkable taxonomic uniqueness. Protracted geographic isolation in the glacial refugium south of the Alps, combined with recurrent demographic bottlenecks, has rendered Canis lupus italicus morphologically, genetically, and genomically differentiated from all other wolf populations worldwide. The subspecific distinctiveness of the Italian wolf was first formally described by Altobello (1921) [13] on the basis of morphological characters, and has since been progressively confirmed by molecular and genomic evidence [14,15]. Genome-wide analyses have revealed the deep evolutionary divergence of southern European wolf lineages from northern European and North American populations, with signatures of prolonged isolation and strong genetic drift consistent with the historical biogeographic scenario of glacial refugium persistence [14,15]. These findings carry direct implications for capture and immobilization practice: the morphological and physiological distinctiveness of C. l. italicus—including its generally smaller body size relative to northern European and North American wolves, and potentially divergent stress response profiles—strengthens the case for developing and validating capture protocols specifically for this subspecies, rather than extrapolating directly from data obtained in other populations. The scale of the Italian recovery has been documented through systematic field surveys and spatially explicit population modelling. A comprehensive national assessment estimated approximately 2388 wolves across 108,534 km^2^ of the Apennine region [11], while subsequent integrated spatial modelling confirmed the continued expansion of the species across south-central Italy [12]. Expanding wolf populations are increasingly colonising human-dominated and peri-urban landscapes [16], a process that intensifies both the demand for scientific monitoring and the complexity of human-wolf coexistence management. This demographic trajectory has progressively increased the need for rigorous research requiring live capture, biological sampling, and telemetry-based monitoring of free-ranging individuals—activities essential for characterising population structure, health status, movement ecology, and connectivity [1,3]. Physical restraint methods, including foothold traps, cable restraints, and Fremont-type humane foot snares, have been widely employed in North American and European wolf management programmes. Among these, the Fremont^®^ humane foot snare has gained increasing acceptance due to its reduced injury profile compared to traditional steel-jaw foothold traps, though comparative welfare data across capture techniques remain limited [17]. Assessment of restraining trap systems requires evaluation of physical, behavioural, and physiological parameters, including injury scoring, signs of distress or exertion during captivity, and indicators of physiological stress [18]. Gese et al. [19] demonstrated that while injury scores between cable restraints and foothold traps may be broadly comparable, capture method can nonetheless influence post-release movement patterns and space use, underscoring the need to evaluate each technique in the context of both immediate physiological impact and longer-term behavioural response. Chemical immobilization in wolves and other large canids has been most extensively studied using alpha-2 adrenergic agonists—particularly medetomidine and xylazine—in combination with dissociative agents such as ketamine, or with cyclohexamine-benzodiazepine combinations such as tiletamine-zolazepam (Telazol^®^) [20]. Medetomidine-ketamine (MK) protocols are widely favoured for their rapid induction, potent sedation, and reversibility via atipamezole [20,21,22]. However, these protocols carry documented risks of hypertension, respiratory depression, and hyperthermia—adverse effects that are particularly relevant under field conditions, where environmental temperature, capture-related stress, and variable body condition interact unpredictably with drug pharmacodynamics [20]. The addition of acepromazine as a phenothiazine adjunct to MK protocols has been proposed to attenuate hypertensive responses and improve muscle relaxation [23]. Its peripheral vasodilatory action, mediated through alpha-1 adrenergic blockade, may furthermore improve peripheral organ perfusion and reduce the risk of capture-related lactic acidosis—a key pathophysiological concern in physically stressed wild mammals. We therefore hypothesized that the MKA combination would reduce the incidence of hyperthermic, hypertensive, and acidotic responses compared to published data on MK protocols alone, while maintaining adequate immobilization quality under operational field conditions. Comprehensive hematological and serum biochemical profiling offers a validated framework for physiological assessment directly translatable to canid field captures [24]. Non-invasive clinical monitoring alone may be insufficient to detect hemodynamic compromise in large wild mammals, emphasising the value of more direct assessment methods where feasible [25]. Despite growing interest in wolf conservation and management in Italy, published data on the physiological responses of free-ranging Apennine wolves to capture and chemical immobilization remain scarce. To our knowledge, no study to date has assessed the combined use of the Fremont™ snare with the MKA protocol in any wolf population worldwide, nor provided a comprehensive suite of physiological and hematobiochemical baseline values for this specific protocol combination under operational field conditions. The present study therefore represents the first systematic evaluation of this approach, reported here for *Canis lupus italicus*. The present study addresses this gap by reporting the physiological and hematobiochemical responses of free-ranging Apennine wolves captured with the Fremont™ snare and immobilized with a standardized MKA protocol, with the aims of evaluating the safety and efficacy of this combined approach and providing baseline reference data for *Canis lupus italicus* relevant to future capture and conservation management programmes.

## 2. Materials and Methods

### 2.1. Study Area and Institutional Framework

This study was conducted within the framework of the LIFE08 NAT/IT/000325 “WOLFNET” project (and post-LIFE actions), a conservation initiative co-funded by the European Commission under the LIFE+ Nature programme, in which GPS radio-collar deployment was specifically directed at supporting direct conservation actions for target wolf packs. The study area comprised the north-western sector of Maiella National Park (Parco Nazionale della Maiella), a protected area of 74,095 hectares established under Italian Law No. 394 of 6 December 1991 and formally delimited by Presidential Decree of 5 June 1995. The park spans 39 municipalities across three provinces of the Abruzzo region—Chieti (27,396 ha), L’Aquila (23,850 ha), and Pescara (22,849 ha)—and encompasses the massifs of the Maiella, Morrone, and Monti Pizzi, with elevations ranging from approximately 400 m to 2793 m a.s.l. at Monte Amaro. The park represents one of the most ecologically significant protected areas in the central Apennines and constitutes a core habitat for the Apennine wolf (*Canis lupus italicus*), as well as for the Marsican brown bear (*Ursus arctos marsicanus*) and the Apennine chamois (*Rupicapra pyrenaica ornata*).

### 2.2. Physical Capture Method

From June 2010 to July 2017, 20 free-ranging Apennine wolves (*Canis lupus italicus*) were captured in Maiella National Park (central Italy) using the Fremont™ Humane Foot Snare. Three chemical immobilization protocols were employed across these 20 capture events; the present study reports exclusively on the 13 individuals immobilized with the medetomidine-ketamine-acepromazine (MKA) protocol; individuals immobilized with alternative protocols (xylazine-tiletamine-zolazepam, *n* = 1; medetomidine-ketamine, *n* = 2) are excluded from the analyses presented here. Full details of the MKA protocol are provided in Section 2.3. It should be noted from the outset that the total sample size (*n* = 13) is inherently limited by the operational and ecological constraints of a long-term field capture programme, and does not permit formal statistical comparisons between subgroups. This limitation is explicitly acknowledged throughout the manuscript and should be considered when interpreting all reported findings. All captures were performed exclusively using the Fremont™ Humane Foot Snare, in compliance with European legislation prohibiting the use of leghold traps. Originally designed for bear capture, the Fremont™ snare was adapted for wolf capture using a 1/8-inch cable (7 × 7 construction: seven wires in seven strands). The device incorporates a mechanical stop preventing complete loop closure, multi-directional swivels allowing rotational movement, and additional springs to absorb shock and minimize trauma to the captured limb. Each trap was equipped with a battery-operated electronic alarm system with GSM communication to ensure rapid response. A dedicated team of four trained personnel—including veterinarians and wildlife biologists—was on standby 24 h per day, 7 days per week, with a target response time not exceeding 35 min from alarm activation to arrival at the capture site. This target was achieved in 12 of 13 captures; in one case (individual F3), a malfunctioning GSM alarm system delayed detection, resulting in an extended interval of approximately 240 min between trap activation and team arrival.

### 2.3. Chemical Immobilization Protocols

Chemical immobilization was conducted year-round, with captures occurring primarily in late spring and autumn under environmental temperatures ranging from 4 °C to 22 °C. Drug delivery was achieved using a CO_2_-powered rifle or blowgun with 3 mL plastic darts fitted with a 1.5 mm diameter × 30 mm length plain needle (Telinject™, Haßmersheim, Germany, or Daninject™, Børkop, Denmark). For juvenile individuals (<1 year of age), chemical immobilization was preceded by physical restraint using net and Y-pole, followed by hand-syringe injection. The injection site was consistently the large muscle mass of the hindquarters.

As noted in Section 2.2, three chemical immobilization protocols were employed across the 20 capture events: (i) a combination of xylazine and tiletamine-zolazepam (XTZ); (ii) medetomidine-ketamine (MK); and (iii) medetomidine-ketamine-acepromazine (MKA) [20,21,22]. The present study reports exclusively on the 13 wolves immobilized with the MKA protocol. Planned doses were medetomidine 50 µg/kg (Domitor^®^, Vetoquinol, Lure, France), ketamine 4 mg/kg (Imalgene^®^, Boehringer Ingelheim, Lyon, France), and acepromazine 0.14 mg/kg (Prequillan^®^, Fatro, Ozzano dell’Emilia, Bologna, Italy), administered as a combined intramuscular injection. Actual doses delivered, calculated on the basis of individual body weight recorded at capture, are reported in the Section 3. Medetomidine immobilization was reversed at the end of the procedure with atipamezole (Antisedan^®^, Vetoquinol, Lure, France) administered intramuscularly at five times the medetomidine dose [20,21,22]. Acepromazine was selected as an adjunct to the MK protocol for its sedative properties, peripheral vasodilatory action through alpha-1 adrenergic blockade, and potential to improve peripheral organ perfusion, attenuate hyperthermia, and reduce the risk of capture-related lactic acidosis [23]. It should be noted that acepromazine is not routinely reversed at the end of the immobilization procedure, as no specific antagonist is available for phenothiazine derivatives; reversal of medetomidine with atipamezole is therefore sufficient to restore autonomous locomotion, with residual acepromazine effects dissipating through hepatic metabolism.

### 2.4. Clinical Monitoring

All immobilization procedures were conducted at or near the trap site, or in a sheltered handling area on a covered pick-up truck during adverse weather conditions. Body weight was determined at the handling site using a digital dynamometer scale. Once loss of consciousness was confirmed, all procedures—including clinical monitoring, blood sampling, biometric measurements, and radio-collar fitting—were conducted with the animal maintained in right lateral or sternal recumbency, as determined by the individual’s clinical condition and logistic requirements at the handling site. Clinical parameters were monitored throughout the immobilization period using a portable multiparameter monitor (iMEC8 Vet Portable Multi-Parameter Veterinary Monitor, Mindray Bio-Medical Electronics, Shenzhen, China) and/or a stethoscope and a digital thermometer, and included heart rate (HR), respiratory rate (RR), peripheral oxygen saturation (SpO_2_) (PM-60 Pulse Oximeter, Mindray Bio-Medical Electronics, Shenzhen, China), and rectal temperature (T). These electronic devices were used in accordance with manufacturer specifications and comply with IEC 60601-1 safety standards. The iMEC8 Vet multiparameter monitor (Mindray^®^) features heart rate accuracy of ±1 bpm or ±1%, and operates within a temperature range of 0–40 °C. The PM-60 pulse oximeter (Mindray^®^) has a manufacturer-stated SpO_2_ accuracy of ±2% (adults/children, 70–100%, stationary) and pulse rate accuracy of ±3 bpm (stationary), operating within the same temperature range. Given the operational constraints of field capture—including variable ambient temperature, animal movement, and peripheral vasoconstriction secondary to alpha-2 adrenergic agonist administration—SpO_2_ readings should be interpreted with awareness of these potential sources of measurement variability, which are inherent to the field application of pulse oximetry in wildlife capture contexts. Physiological parameters were recorded at minimum three times per animal: T1: within 15 min of complete induction, i.e., the finding of loss of consciousness/the moment of starting the handling; T2: within the following 15 min; T3: within the subsequent 15 min or immediately before antagonist administration. In two individuals, active thermoregulatory interventions were applied in response to clinically significant thermal deviations: individual M1, a juvenile presenting hypothermia (rectal temperature 34.9 °C at T1), was treated with a thermal blanket and direct heat sources; individual F4, a pregnant female presenting hyperthermia (rectal temperature 41.4 °C at T1), was treated with ice packs and cool water aspersion on the coat. In both cases, values recorded at subsequent time points (T2, T3) reflect the response to these interventions rather than the spontaneous pharmacological course of immobilization, and should be interpreted accordingly. Statistical summaries are based on per-animal means, each representing the mean of three recordings obtained at these standardized intervals. All individual values were retained in the analysis. Condition-specific clinical findings observed in individual animals are discussed separately in the relevant sections. For SpO_2_, only values recorded prior to supplemental oxygen administration were included in the analysis; oxygen supplementation was performed in four individuals (M1, F4, M5, F7), in all cases after 30 min from induction. All wolves were fitted with GPS/GSM radiocollars (Followit^®^, Lindesberg, Sweden, or Vectronic^®^, Berlin, Germany), programmed with an intensive monitoring protocol to allow continuous assessment of animal health status, activity, and survival in the post-capture period. For each captured individual, the minimum confirmed post-capture survival period was determined through continuous GPS/GSM radiocollar tracking. In cases where collar functionality ceased prior to the end of the monitoring period, survival was further documented through alternative methods, including field recovery of injured (individual F6) or deceased animals, and detection via camera trapping. This multi-method approach allowed the establishment of minimum survival periods for all 13 individuals regardless of collar operational status.

### 2.5. Hematological, Biochemical, Venous Blood Gas and Serological Analyses

Blood samples were collected from the jugular or cephalic vein during immobilization and processed within 24 h of collection at the IZSAM Central Laboratory (Istituto Zooprofilattico Sperimentale Abruzzo e Molise—Teramo, Italy). Complete blood count (CBC) was performed on whole blood samples using a cytochemical automated analyser (procedure IZS TE S5 B2.1.6 SOP003-Rev0-07). Parameters assessed included white blood cell count (WBC), red blood cell count (RBC), haemoglobin concentration (HGB), haematocrit (HCT), mean corpuscular volume (MCV), mean corpuscular haemoglobin (MCH), mean corpuscular haemoglobin concentration (MCHC), red cell distribution width (RDW), haemoglobin distribution width (HDW), platelet count (PLT), mean platelet volume (MPV), and differential leukocyte count (neutrophils, lymphocytes, monocytes, eosinophils, basophils, and large unstained cells, LUC). A core panel of serum biochemical parameters was analysed at IZSAM according to procedure IZS TE S5 B2.1.6 SOP001-Rev1-07. Aspartate aminotransferase (AST/GOT), gamma-glutamyl transferase (GGT), alanine aminotransferase (ALT/GPT), amylase, and alkaline phosphatase (ALP) were measured by kinetic assay; blood urea nitrogen (BUN) and glucose by enzymatic assay; uric acid, cholesterol, and creatinine by enzymatic-colorimetric assay; triglycerides by kinetic-colorimetric assay; total protein, albumin, total bilirubin, and calcium by colorimetric assay. Additional biochemical parameters—specifically globulins (GLOB), phosphorus (PHOS), potassium (K^+^), and sodium (Na^+^)—were analysed at the Wildlife Research Center laboratory of Maiella National Park using the Vetscan^®^ VS2 analyzer (version 2.1.5; Zoetis, formerly Abaxis, Inc., Union City, CA, USA; Comprehensive Diagnostic Profile rotor #500-0038). Venous blood gas analysis was performed opportunistically during immobilization in six individuals (F2, M3, M4, F4, M6, F7), in all cases using a handheld blood gas analyser (i-STAT 1^®^, Abbott Laboratories, Abbott Park, IL, USA) with CG4+ and EC8+ cartridges. Arterial blood sampling was not feasible under the logistic constraints of field capture operations; venous samples were therefore obtained as the available alternative. Parameters reported include venous pH, lactate, pCO_2_, HCO_3_^−^, and base excess (BE). The i-STAT 1^®^ handheld analyser was operated in accordance with manufacturer guidelines. CG4+ and EC8+ cartridges were stored refrigerated (2–8 °C) and equilibrated to ambient temperature immediately prior to use, as required by the manufacturer. The analyser itself was maintained within the recommended operating temperature range (16–30 °C); under field conditions, this required active temperature management of the device, including warming in cold weather. Venous blood gas analysis was performed immediately after blood collection to minimise pre-analytical variability. The field application of this analyser in free-ranging wolves represents, to our knowledge, a novel use of point-of-care blood gas technology in this species under operational capture conditions; the data presented should therefore be regarded as preliminary, obtained under variable field conditions that did not always meet the optimal analytical requirements for this device. Creatine kinase (CK) was not included in the analytical panel of this study; in cases where muscular involvement was suspected on clinical grounds, myopathy was therefore inferred from the combination of markedly elevated AST and ALT activities, clinical signs, and contextual capture parameters, in the absence of direct CK measurement. This represents a limitation of the present dataset, which should be addressed in future capture studies. In order to evaluate potential pathogen exposure that could influence the health status of captured individuals, serological analyses were performed on 12 of 13 wolves. Antibodies against Canine Protoparvovirus 1 (CPV), Canine Adenoviruses (CadVs), Canine Distemper Virus (CDV), and Canid alphaherpesvirus 1 (CHV) were measured as previously described [24,25]. Serological testing for *Leptospira* spp. was performed using the microscopic agglutination test (MAT), targeting a panel of serovars including Australis and Bratislava. *Ehrlichia* spp. serology was also included in the panel.

### 2.6. Age Determination

Age classification was determined through morphological evaluation of tooth replacement, wear patterns, and overall physical development conducted while each animal was fully sedated during field processing [26]. This approach reliably discriminates between pups (<1 year) and adults (≥1 year). For individuals aged ≥ 1 year, monthly age estimates (e.g., 18, 24, 48 months) are also provided; however, these finer estimates are based solely on morphological observation and general growth characteristics, without the support of cementum annuli validation. Consequently, these adult monthly age estimates must be considered provisional field assessments rather than precise determinations. For pups (<1 year), monthly age estimates (e.g., 6, 8, 12 months) are based on the known birth seasonality of wolves in the Northern Hemisphere, with parturition typically occurring between April and May [27]. In the Apennines, this pattern is consistent with documented Italian wolf reproductive biology [28]. Capture date relative to this expected birth peak allowed approximation of age in months for individuals aged less than one year. Cementum annuli analysis was not performed; therefore, all age estimates provided must be regarded as provisional and based exclusively on field assessments. A potential source of observer bias inherent to morphological age estimation should be acknowledged: all assessments were performed by the attending veterinarian at the time of capture, without independent verification by a second observer. While the binary discrimination between juveniles (<1 year) and adults (≥1 year) is considered reliable on the basis of well-established morphological criteria [26], the finer monthly age estimates for adult individuals carry a degree of subjectivity that cannot be fully quantified in the absence of cementum annuli validation. Users of the age data presented in Table 1 should interpret adult monthly estimates accordingly.

### 2.7. Physical Examination and Injury Assessment

Physical examination was performed on each immobilized individual immediately following induction of anaesthesia. Foot injury severity was assessed using the modified trap injury scoring system described by Kuehn et al. (1986) [29], which classifies injuries on a graduated scale from Grade 0 (no lesion) to Grade 4 (severe injury with skeletal or tendinous involvement). The presence of oral mucosal lesions and reversible distal limb oedema were recorded as additional indicators of restraint-related trauma. All examinations were performed by the attending veterinarian at the time of capture.

### 2.8. Post-Capture Monitoring

All wolves were fitted with GPS/GSM radiocollars (Followit^®^, Lindesberg, Sweden, or Vectronic^®^, Berlin, Germany), programmed with an intensive monitoring protocol to allow continuous assessment of animal health status, activity, and survival in the post-capture period. The GPS/GSM radiocollar data served a dual purpose: supporting the conservation objectives of the LIFE WOLFNET project through characterisation of space use, movement ecology, and pack dynamics, and providing post-capture health and survival monitoring for each immobilized individual. For each captured individual, the minimum confirmed post-capture survival period was determined through continuous GPS/GSM radiocollar tracking. In cases where collar functionality ceased prior to the end of the monitoring period, survival was further documented through alternative methods, including field recovery of injured or deceased animals and detection via camera trapping. This multi-method approach allowed the establishment of minimum survival periods for all 13 individuals regardless of collar operational status.

The minimum post-capture monitoring period was not pre-defined as a fixed study endpoint; the values reported in Table 1 therefore represent the empirically confirmed minimum survival periods for each individual, ranging from 4 to 57 months across the cohort.

### 2.9. Ethical Statement

All capture and immobilization procedures were carried out under authorization from the Italian Ministry of Environment (permit No. 026416, 22 June 2009, extended by permit No. 46090, 2014), issued pursuant to Presidential Decree No. 357/1997, which transposes Council Directive 92/43/EEC (Habitats Directive) into Italian national law. Ministerial authorization was granted on the basis of a formal technical opinion issued by ISPRA (Istituto Superiore per la Protezione e la Ricerca Ambientale), the Italian national authority competent for wildlife conservation and research. In the Italian regulatory framework applicable during the study period, the ISPRA technical opinion encompasses both conservation and animal welfare considerations, and is functionally equivalent to the Institutional Animal Care and Use Committee (IACUC) approval or Institutional Review Board (IRB) statement required in other national jurisdictions.

All procedures were conducted in accordance with current Italian and European legislation on the protection of wildlife, and in compliance with Council Regulation (EEC) No. 3254/91 of 4 November 1991, which prohibits the use of leghold traps within the European Union and establishes humane trapping standards for species listed in Annex I of that Regulation, within which the grey wolf (*Canis lupus*) is included.

### 2.10. Data Availability and Use of Generative AI

The dataset supporting the results of this study is available upon request from the Wildlife Research Center of Maiella National Park—Veterinary Service (simone.angelucci@parcomajella.it). In the preparation of this manuscript, the authors used Claude (Anthropic, claude.ai) to assist in English translation, scientific writing, and formatting. All content was reviewed, verified, and validated by the authors, who take full responsibility for the accuracy and integrity of the published work.

### 2.11. Statistical Analysis

All data are presented as descriptive statistics: continuous variables are expressed as mean ± standard deviation (SD) and range; categorical variables are expressed as absolute frequencies and percentages. No inferential statistical tests were applied, as the overall sample size (*n* = 13) and the composition of subgroups—in particular the juvenile cohort (*n* = 3)—do not permit hypothesis testing with adequate statistical power. This study is therefore strictly observational and descriptive in design.

Missing data were handled as follows: individual F6 was excluded from hematological, biochemical, and serological analyses due to the impossibility of blood sample collection at the time of capture (technical and logistical constraints); this exclusion is noted explicitly in each relevant Section 3, and all analyses involving blood parameters are based on *n* = 12. Venous blood gas analysis was available for six individuals only (*n* = 6), owing to a combination of logistical constraints and suboptimal field conditions; this subset is identified by individual in Section 2.5 and Section 3.7. All descriptive statistics were calculated using Microsoft Excel (Microsoft Corporation, Redmond, WA, USA).

## 3. Results

### 3.1. Animal Demographics and Capture Conditions

Thirteen free-ranging Apennine wolves (Canis lupus italicus) were captured and immobilized with the MKA protocol between June 2010 and July 2017 in Maiella National Park. The cohort comprised 6 males and 7 females, of which 10 were adults (4 males, 6 females) and 3 were juveniles (2 males, 1 female). Mean body weight was 29.52 ± 5.22 kg (range: 22.0–38.3 kg) in adults and 16.60 ± 2.92 kg (range: 13.5–19.3 kg) in juveniles. Age at capture ranged from 6 to 48 months; all age estimates are based on field morphological assessment as described in Section 2.6. Individual capture data, including age at capture, date, body weight, and minimum confirmed post-capture survival period, are reported in Table 1.

Individual F4, captured on 8 April 2013 at an estimated age of 48 months, presented clear clinical signs of advanced pregnancy at the time of handling, including abdominal distension and visibly increased respiratory effort consistent with uterine compression of the diaphragm (see Supplementary Material S5). The mean time elapsed between trap activation and drug administration was 78.00 ± 53.95 min (range: 36–240 min). The extended maximum interval of 240 min was recorded for individual F3, in which a malfunctioning GSM alarm system delayed detection until the routine safety inspection; despite this prolonged pre-intervention interval, clinical assessment of F3 at the time of handling did not reveal parameters indicative of critical physiological compromise. With the exception of one adult female that required a second dart (F5), all individuals were fully immobilized following a single intramuscular injection.

### 3.2. Anesthetic Protocol and Drug Doses

Planned doses were medetomidine 50 µg/kg, ketamine 4 mg/kg, and acepromazine 0.14 mg/kg. Mean doses actually delivered are reported in Table 2. Adults received medetomidine 0.052 ± 0.005 mg/kg, ketamine 4.400 ± 0.474 mg/kg, and acepromazine 0.142 ± 0.037 mg/kg. Juveniles received medetomidine 0.059 ± 0.016 mg/kg, ketamine 3.989 ± 0.824 mg/kg, and acepromazine 0.149 ± 0.040 mg/kg. In juvenile individuals, chemical immobilization was preceded by physical restraint using net and Y-pole, followed by hand-syringe injection; no complications directly attributable to this procedure were recorded.

### 3.3. Physiological Parameters During Immobilization

The first clinical sign of drug effect was loss of head posture (“head down”), occurring at a mean of 8.60 ± 2.95 min in adults and 6.00 ± 2.00 min in juveniles. Full induction was reached at a mean of 17.30 ± 6.36 min in adults and 10.00 ± 5.29 min in juveniles. Physiological parameters recorded during immobilization are summarized in Table 3, stratified by age class. Mean respiratory rate was 24.5 ± 14.1 breaths/min in adults and 20.8 ± 6.6 breaths/min in juveniles (cohort mean: 24 ± 13 breaths/min, range: 13–60). Mean heart rate was 103.1 ± 14.0 bpm in adults and 89.6 ± 8.2 bpm in juveniles (cohort mean: 100 ± 15 bpm, range: 84–126 bpm). Mean body temperature was 38.5 ± 1.1 °C in adults and 37.1 ± 1.5 °C in juveniles (cohort mean: 38.1 ± 1.3 °C, range: 36.0–41.0 °C). Mean SpO_2_ was 88.8 ± 8.5% in adults (*n* = 9) and 84.2 ± 16.2% in juveniles (cohort mean: 88 ± 11%, range: 66–97%; *n* = 12). No formal statistical comparison between age classes was performed. The juvenile subgroup comprised only three individuals (*n* = 3), a sample size that precludes any inferential analysis with adequate statistical power; the overall cohort size (*n* = 13) similarly limits the generalisability of the findings, a constraint inherent to field-based capture studies of large carnivores. Individual values for all parameters at each recorded time point are provided in Supplementary Material S7. No case of clinically significant hyperthermia requiring active cooling was recorded in the cohort as a whole. Two individuals presented condition-specific clinical findings requiring targeted intervention: individual F4, the pregnant female, exhibited the highest respiratory rate (60 breaths/min) and heart rate (126 bpm) in the cohort, and presented hyperthermia (T1: 41.4 °C) managed with ice packs and cool water aspersion; individual M1, the smallest juvenile (13.5 kg, captured in January), presented hypothermia (T1: 34.9 °C) managed with thermal blanket and direct heat sources. In both cases, subsequent temperature recordings reflect the response to these interventions. Supplemental oxygen was administered to four individuals (M1, F4, M5, F7) following SpO_2_ values recorded prior to supplementation; only pre-supplementation values are included in the analysis.

### 3.4. Results of Physical Examination and Injury Assessment

Physical examination was performed on all 13 immobilized individuals immediately following induction of anaesthesia. Results are summarized according to the modified trap injury scoring system of Kuehn et al. (1986) [29]. A total of 61.5% of wolves (8/13) showed no detectable foot lesions (Grade 0). The remaining 38.5% (5/13) exhibited superficial excoriations without deep tissue involvement: three individuals presented Grade 1 lesions (superficial abrasions, total extent < 1 cm; see Supplementary Material S3) and two individuals presented Grade 2 lesions (superficial cuts, total extent < 2.5 cm; see Supplementary Material S4). No skeletal or tendinous injuries were recorded in any individual. Reversible distal limb oedema was observed in 30.8% of individuals (4/13). Minor oral mucosal lesions were noted in 46.2% of individuals (6/13), attributable to snare-directed behaviour prior to chemical immobilization.

### 3.5. Recovery Timeline

Atipamezole was administered intramuscularly at 0.4 mg/kg at a mean of 75.90 ± 16.15 min post-induction in adults and 62.67 ± 15.50 min post-induction in juveniles. No reversal agent was administered for ketamine or acepromazine; as noted in Section 2.3, no specific antagonist is available for phenothiazine derivatives, and residual acepromazine effects dissipate through hepatic metabolism following atipamezole-mediated recovery from medetomidine. In juvenile individuals, voluntary head movements were first observed at a mean of 52.00 ± 19.36 min post-induction, in some cases prior to atipamezole administration. In adults, voluntary head movements were first observed at a mean of 79.30 ± 19.84 min post-induction. Return to stable four-legged posture occurred at a mean of 97.40 ± 27.80 min in adults and 75.33 ± 23.69 min in juveniles. Autonomous departure from the handling site was recorded at a mean of 108.90 ± 33.14 min in adults and 87.33 ± 29.48 min in juveniles. No fatalities or severe adverse events occurred during immobilization or recovery in any of the 13 individuals.

### 3.6. Hematological Parameters

Blood samples for hematological analysis were available from 12 of 13 immobilized wolves. Samples could not be collected from individual F6 due to technical and logistical constraints at the time of capture. Results are summarized in Table 3. Mean values for the cohort (*n* = 12): RBC 7.0 ± 1.1 × 10^12^/L, haemoglobin 175.1 ± 26.2 g/L, haematocrit 47.8 ± 9.0%, MCV 68.8 ± 5.7 fl, MCH 25.1 ± 3.3 pg, MCHC 370.1 ± 69.9 g/L, RDW 20.0 ± 8.7%, HDW 300.4 ± 103.0 g/L, platelets 244.5 ± 111.2 × 10^9^/L, MPV 9.8 ± 3.0 fl, WBC 16.0 ± 5.4 × 10^9^/L. Differential counts showed neutrophil predominance (73.2 ± 12.2%), with lymphocytes 14.6 ± 7.5%, monocytes 6.9 ± 6.6%, eosinophils 6.4 ± 3.8%, basophils 0.8 ± 0.6%, and large unstained cells (LUC) 0.2 ± 0.2%. No stress leukogram, significant anaemia, or thrombocytopenia was identified in the cohort as a whole. Two individual findings warrant specific reporting. Individual F7 presented haematocrit of 68.2%, consistent with hemoconcentration. Two individuals presented MCHC values at the upper extreme of the cohort range (up to 492 g/L) and HDW values up to 550 g/L; these findings may reflect hemoconcentration or analytical variability under field conditions, and are noted for completeness. No formal statistical comparison between age classes or sexes was performed given the limited subgroup sizes.

### 3.7. Serum Biochemistry and Venous Blood Gas Analysis

Serum biochemical samples were available from 12 of 13 individuals (individual F6 excluded, as noted in Section 3.6). Results are summarized in Table 4. Mean total protein was 68 ± 10 g/L, albumin 31 ± 6 g/L, globulins 47 ± 14 g/L. Hepatic enzyme activities: ALT 79 ± 56 IU/L (range: 26–195 IU/L), AST 161 ± 177 IU/L (range: 52–699 IU/L), ALP 92 ± 49 IU/L. Mean creatinine 79.23 ± 28 µmol/L, BUN 12.48 ± 6 mmol/L, glucose 5.27 ± 2 mmol/L. No formal statistical comparison between age classes or sexes was performed given the limited subgroup sizes. Two individuals presented condition-specific biochemical findings. Individual F4, an adult female in advanced pregnancy at the time of capture, exhibited elevated total protein (90 g/L), markedly reduced albumin (16 g/L), ALT 52 IU/L, and AST 68 IU/L. Individual F7, a 14-month-old female, presented markedly elevated AST (699 IU/L) and ALT (195 IU/L), together with the haematocrit of 68.2% noted in Section 3.6. Creatine kinase (CK) was not included in the analytical panel of this study; muscular involvement in F7 was therefore assessed in the absence of direct CK measurement.

Venous blood gas analysis was performed opportunistically in six individuals (F2, M3, M4, F4, M6, F7); results are reported in Supplementary Material S8. Venous pH ranged from 7.221 to 7.429 (mean ± SD: 7.36 ± 0.09). Four individuals (F2, M3, M4, M6) presented pH values within or immediately adjacent to the canine venous reference range (7.35–7.45) [26]. Two individuals (F4 and F7) presented pH values below 7.30 (7.274 and 7.221, respectively). Venous lactate ranged from 1.02 to 3.49 mmol/L (mean ± SD: 1.83 ± 1.19 mmol/L). Four individuals (F2, M3, M4, M6) showed lactate values within the reference range (0.43–2.10 mmol/L) [30], consistent with values previously reported in healthy dogs (<2.5 mmol/L and <2.6 mmol/L, respectively) [31,32]. The two individuals with lower pH values (F4 and F7) also presented the highest lactate concentrations (3.23 and 3.49 mmol/L, respectively). pCO_2_ ranged from 10.7 to 61.2 mmHg; the value recorded in F2 (10.7 mmHg) is anomalous and inconsistent with the concurrent venous parameters of that individual (pH 7.429, HCO_3_^−^ 18.7 mmol/L), and a technical or sampling artefact cannot be excluded; this value is retained in Supplementary Material S8 for transparency but excluded from the pCO_2_ mean calculation (mean pCO_2_ *n* = 5:51.4 ± 7.6 mmHg). Mean HCO_3_^−^ was 23.3 ± 5.3 mmol/L and mean base excess −2.2 ± 6.0 mmol/L. Individual M3 presented a base excess of +8 mmol/L with elevated HCO_3_^−^ (32.5 mmol/L), in the context of a normal pH (7.397).

### 3.8. Serological Analyses

Serological investigations were carried out on 12 of 13 individuals (individual F6 excluded, as noted in Section 3.6). Ten animals were seropositive for canine distemper virus (CDV) with antibody titres ranging from 1:4 to ≥1:256. Nine animals were seropositive for canine adenoviruses (CadVs) with antibody titres ranging from 1:4 to ≥1:256. Eight animals were seropositive for Canine Protoparvovirus 1 (CPV) with antibody titres ranging from 1:16 to 1:256. Three animals were seropositive for Canid Alphaherpesvirus 1 (CHV) with antibody titres ranging from 1:8 to 1:32. Antibodies against *Leptospira* spp. were detected in two individuals, with titres of 1:100 and 1:200 for the *australis* and *bratislava* serovars respectively. All seropositive individuals were clinically asymptomatic at the time of capture. No animal tested seropositive for *Ehrlichia* spp.

### 3.9. Post-Capture Survival and Preliminary GPS Observations

All 13 immobilized individuals survived the capture procedure. Minimum confirmed post-capture survival periods, determined through GPS/GSM radiocollar tracking and alternative monitoring methods as described in Section 2.8, ranged from 4 to 57 months across the cohort and are reported in Table 1. Preliminary GPS tracking data were available for three individuals (M2, F2, F3) in the immediate post-release period. In all three cases, active displacement from the capture site ceased within approximately two hours of release, after which the animals alternated brief relocations with extended stationary periods. The ratio of active to total GPS fixes in the first 48 h post-release was consistently low across individuals (19/96 for M2; 15/48 for F2; 33/96 for F3). These observations are preliminary and descriptive in nature; given the very limited sample size (*n* = 3), no statistical inference can be drawn. A dedicated analysis of post-release movement data for the full cohort will be presented in a separate publication.

## 4. Discussion

### 4.1. Capture Method and Pre-Induction Stress Management

The Fremont™ Humane Foot Snare proved effective for the capture of both adult and juvenile Apennine wolves, confirming its applicability across age classes under operational field conditions. The device was used with a 1/8-inch cable (7 × 7 construction), mechanical stop, multi-directional swivels, and shock-absorbing springs; these technical specifications are relevant to the reproducibility of the injury profile described in Section 3.4 and should be replicated in future applications of this protocol. The mean time elapsed between trap activation and drug administration was 78.00 ± 53.95 min. As noted in Section 3.1, this interval does not reflect the response time to the trap site—which was ≤35 min in 12 of 13 captures—but rather the total duration of the multi-step operational sequence following arrival: visual assessment of the restrained animal, body weight estimation, drug dose calculation, approach, and—depending on age class—dart delivery or mechanical restraint with net and Y-pole followed by hand injection in juveniles. The extended interval recorded for individual F3 (240 min), attributable to a GSM alarm malfunction, did not result in critical physiological compromise, suggesting a degree of resilience in the species to prolonged conscious restraint when physical trauma is minimised by appropriate snare design and trap placement. Direct observation via camera traps positioned at capture sites revealed a characteristic behavioural pattern: following initial restraint, wolves engaged in organised, goal-directed escape attempts but remained relatively calm during the period preceding the arrival of the capture team (see Supplementary Material S2). A marked increase in behavioural stress was consistently observed at the moment wolves detected the approaching vehicle and operators, with the peak stress response occurring during the final approach phase. This field observation is consistent with the hypothesis that the dominant driver of capture-associated physiopathology in wild mammals is not the duration or intensity of physical exertion *per se*, but rather the psychological component of the stress response—specifically, the perception of an approaching threat [20,23]. This interpretation is supported by comparative data from Meyer et al. (2008) [33] in impalas, where the magnitude of hyperthermia during chemical immobilization was determined primarily by the psychological fright response rather than by drug pharmacology. It should be noted, however, that impalas are prey species and wolves are apex predators; their neuroendocrine stress responses may differ substantially, and this comparison should therefore be regarded as a supporting hypothesis rather than conclusive evidence. The practical implication for field operations is clear: minimising the duration and intensity of anthropogenic stimulation from first detection by the animal through to complete anaesthetic induction is the most important welfare intervention available to the capture team. Based on our experience, the approach must be immediate and decisive, drug delivery rapid and efficient, and operators must withdraw as soon as possible after injection, minimising visual and auditory stimuli during the induction phase. The use of night-vision equipment to monitor induction at distance is recommended where feasible, and repeated approach attempts before 15 min post-injection should be avoided [17,20,23].

### 4.2. Anesthetic Protocol: Induction, Physiological Parameters, and the Role of Acepromazine

Mean induction times of 17.30 ± 6.36 min in adults and 10.00 ± 5.29 min in juveniles are consistent with published data for MK-based protocols in free-ranging gray wolves and other large canids [20]. The shorter induction times in juveniles likely reflect differences in body mass, metabolic rate, and—in this cohort—the route of administration, given that juveniles received hand-syringe injection following physical restraint rather than remote dart delivery. In juvenile individuals, voluntary head movements were observed prior to atipamezole administration in some cases, consistent with more rapid pharmacokinetic clearance expected in younger and lighter individuals. The potential contribution of pre-injection physical restraint stress to the physiological parameters recorded in juveniles cannot be excluded and should be considered when interpreting age-class differences in this cohort. The MKA protocol produced stable anaesthesia with no cases of clinically significant hyperthermia in the cohort as a whole. The maximum individual body temperature recorded was 41.4 °C in individual F4; as discussed in Section 3.3, this value preceded active cooling intervention and is attributable to the combined effects of advanced pregnancy and capture stress rather than to a primary pharmacological adverse effect. Drew and Struthers (2025) [34], in a retrospective analysis of 490 captures in Idaho, demonstrated that ketamine-medetomidine was associated with hyperthermia (≥41 °C) in 13.7% of summer and 26.7% of winter aerial captures. Initial hyperthermia was also documented in captive red wolves anaesthetized with MKA by Sladky et al. (2000) [22]. The mean body temperature recorded in our cohort (38.1 ± 1.3 °C, range: 36.0–41.0 °C) was notably lower than these published values, and well below the hyperthermia threshold of 41 °C at cohort level. Two elements are likely to have contributed to this favourable thermal profile. First, the strict field management protocol—immediate approach, rapid drug delivery, and prompt withdrawal of personnel during induction—was specifically designed to minimise psychoemotional stimulation, recognised as a primary driver of stress-induced hyperthermia in captured wildlife [33]. Second, the peripheral vasodilatory properties of acepromazine, through alpha-1 adrenergic blockade, may have promoted cutaneous heat dissipation and improved peripheral organ perfusion, potentially reducing the risk of capture-related lactic acidosis. The relative contribution of each factor cannot be determined from the present dataset. Arterial blood pressure was not measured in this study; the proposed attenuation of acepromazine on hypertensive responses therefore remains unproven under the field conditions described, and represents a limitation of the present dataset. Future capture protocols should incorporate non-invasive blood pressure assessment where logistically feasible [35]. The use of medetomidine carries an inherent risk of ventilation-perfusion mismatch through pulmonary vasoconstriction and altered regional blood flow distribution. Mean SpO_2_ of 88 ± 11% (range: 66–97%; *n* = 12) indicates that hypoxaemia was present in a subset of individuals, consistent with the V/Q mismatch expected with medetomidine-based immobilisation. Supplemental oxygen was administered to four individuals (M1, F4, M5, F7) following SpO_2_ values recorded prior to supplementation. The absence of arterial blood gas capability in the field precluded assessment of PaO_2_, PaCO_2_, and derived parameters such as alveolar-arterial oxygen gradient; acquisition of portable arterial blood gas capability is strongly recommended for future wildlife capture protocols. The cardiorespiratory data from Sladky et al. (2000) [22] in captive red wolves provide the most directly comparable published reference for our MKA cohort. Heart rates in the MKA group in that study ranged from 87.6 to 102.4 bpm across measurement intervals, values closely comparable to our cohort mean of 100 ± 15 bpm. Comparative data from Talukdar and Raina (2023) [36] on Tibetan wolves and Gutema et al. (2018) [37] on African wolves further confirm the importance of thermal and cardiorespiratory monitoring in all alpha-2-ketamine field protocols.

### 4.3. Hematological and Biochemical Parameters: Baseline Reference Values and Individual Cases

To our knowledge, this study provides the first systematic report of hematological and serum biochemical reference values for free-ranging Apennine wolves (*Canis lupus italicus*) captured with a humane foot snare. The values obtained were broadly consistent with published reference data for gray wolves from southcentral Alaska [38], Minnesota and Alaska [39], Scandinavia [40], captive wolves [22], and Iberian wolves captured with leghold snares [41]. It should be noted that direct comparisons between studies are limited by differences in capture method, anaesthetic protocol, geographic population, and laboratory methodology; the values reported here are therefore best interpreted as population-specific baseline data for *C. l. italicus* under the specific conditions described, rather than as universal reference intervals for the species. Hematological findings did not reveal stress leukograms, significant anaemia, or thrombocytopenia in the majority of individuals. The interpretive value of the absence of a stress leukogram requires contextual qualification. Two distinct leukocyte responses to stress must be distinguished: the rapid catecholamine-mediated physiologic leukocytosis, which develops within minutes and resolves within 30–60 min, and the corticosteroid-mediated stress leukogram, which requires 4–8 h to develop [42,43,44]. Neither response would be expected to be detectable at the time of sampling in this study: the catecholamine response had already resolved following anaesthetic induction, and the corticosteroid-mediated pattern had not yet had sufficient time to develop within the mean handling duration. The absence of leukogram changes therefore reflects the sampling timeframe rather than the absence of a stress response per se. The elevated WBC (cohort mean 16.0 ± 5.4 × 10^9^/L) with neutrophil predominance (73.2%) observed in this cohort is consistent with the physiological leukocytosis reported in free-ranging wolves under field capture conditions [39,41], and does not in itself constitute a stress leukogram in the absence of a concurrent lymphopenia and eosinopenia pattern. The absence of a non-manipulated control group—whether captive wolves or individuals sampled by non-invasive means—limits the interpretation of these findings, and is acknowledged as a structural limitation of field-based capture studies of this kind. Two individuals with markedly elevated MCHC (up to 492 g/L) and HDW (up to 550 g/L) values were identified. These findings may reflect hemoconcentration secondary to dehydration or physical exertion, or may represent analytical variability under field conditions affecting sample quality; both interpretations are consistent with the capture context and neither is indicative of a primary haematological disorder. Similarly, the wide range in platelet counts (131–526 × 10^9^/L) likely reflects a combination of physiological variation and platelet clumping artefact, a known source of variability in field-collected canid samples. Individual F4, an adult female in advanced pregnancy at the time of capture (see Supplementary Material S5), exhibited the highest respiratory rate (60 breaths/min) and heart rate (126 bpm) in the cohort, consistent with the physiological adaptations expected in mid-to-late gestation: progressive uterine distension reduces functional residual lung capacity, increases respiratory work, and elevates cardiac output requirements. The biochemical profile of F4—elevated total protein with markedly reduced albumin (16 g/L)—is consistent with gestational haemodilution-associated hypoalbuminaemia documented in dogs from mid-pregnancy onward [45,46]. The venous blood gas values recorded in F4 (pH 7.274, lactate 3.23 mmol/L, BE −6 mmol/L) represent the most marked acid-base deviation in the cohort, attributable to the combined physiological demands of capture stress and advanced pregnancy. By current definitions, these values fall within the range of mild to moderate hyperlactataemia with concurrent mild acidaemia, and remain below the thresholds defining lactic acidosis. Critically, post-capture GPS monitoring confirmed that F4 successfully delivered and raised four pups in the subsequent summer, demonstrating the absence of lasting reproductive impact from the capture event. When managing pregnant females with this protocol, several precautionary adaptations should be considered: minimising handling time to reduce the cumulative physiological burden; positioning the animal in left lateral rather than right lateral recumbency where possible to reduce aortocaval compression by the gravid uterus; and maintaining particular vigilance for signs of respiratory compromise given the reduced functional residual capacity associated with advanced gestation. These recommendations are consistent with published guidance on anaesthesia in pregnant mammals [20]. Individual F7, a 14-month-old female, presented haematocrit of 68.2% and markedly elevated AST (699 IU/L) and ALT (195 IU/L), findings consistent with hemoconcentration secondary to dehydration and transient capture-related myopathy. Creatine kinase (CK) was not included in the analytical panel of this study; myopathy was therefore inferred from the combination of markedly elevated transaminase activities, haemoconcentration, and the clinical context of capture stress, in the absence of direct CK measurement. This represents a limitation of the present dataset. The pattern of enzyme elevation in the absence of other systemic abnormalities is consistent with a self-limiting, capture-stress-mediated event, as previously documented in free-ranging canids [34,39,41]. Post-capture GPS monitoring confirmed full recovery of normal ranging behaviour within the subsequent monitoring period. Venous blood gas data indicate that frank acidosis did not develop in the majority of assessed individuals. Four of six wolves presented pH values consistent with physiological compensation. Individual M3 presented a base excess of +8 mmol/L with elevated HCO_3_^−^ (32.5 mmol/L) in the context of a normal pH (7.397), a pattern consistent with compensatory metabolic alkalosis secondary to medetomidine-induced hypoventilation and resultant hypercapnia (pCO_2_ 52.8 mmHg). No cardiovascular complications were recorded in any individual, suggesting that the risk factors identified by Mattaliano et al. (2023) [47]—prolonged anaesthetic time and sustained α2-adrenoceptor agonist exposure—were not operative under the brief field immobilisation conditions described here. Serological findings indicate previous exposure to common canid pathogens—principally CDV, CadVs, and CPV—consistent with patterns reported in other free-ranging wolf populations [48,49,50]. All seropositive individuals were clinically asymptomatic at the time of capture; in the absence of clinical signs or virus isolation, seropositivity indicates prior exposure rather than active disease. The limited seropositivity for Canid alphaherpesvirus 1 and for Leptospira Australis and Bratislava serovars suggests occasional exposure to both pathogens. In the absence of clinically relevant signs in any individual under study, the influence of these infections on the health status of the captured animals can be considered negligible. Regarding Leptospira serology specifically, the detection of Australis and Bratislava serovars is consistent with the known distribution of these serovars in Italian and European wildlife [51,52] even though, to our knowledge, no evidence of these serovars has previously been reported in the Apennine wolf. Given the small sample size, these findings should be interpreted as indicators of pathogen circulation in the study area rather than as evidence of population-level health impact. The following limitations of the present dataset are explicitly acknowledged: small overall sample size (*n* = 13); absence of arterial blood gas measurement; absence of blood pressure monitoring; absence of a control group of non-manipulated or differently captured individuals; absence of CK measurement (Creatine kinase (CK) was not included in the analytical panel of this study, as neither the IZSAM standard biochemical procedure (IZS TE S5 B2.1.6 SOP001-Rev1-07) nor the Vetscan^®^ VS2 Comprehensive Diagnostic Profile rotor (#500-0038) employed in this study includes CK as a standard parameter, retrospective addition of CK analysis was not feasible given sample storage constraints); and post-release GPS data available for only 3 individuals. Venous blood gas data available for only 6 of 13 individuals; this represents the first reported field application of a handheld point-of-care blood gas analyser in free-ranging wolves under operational capture conditions. Successful analysis required the simultaneous availability of adequate field conditions—including maintenance of the i-STAT 1^®^ analyser and cartridges within their operational temperature ranges, appropriate terrain for correct sample handling, and sufficient sample quality—conditions that were not consistently achievable across all 13 captures. The venous blood gas data reported here therefore carry an additional layer of interpretive caution beyond the small sample size: they are best regarded as preliminary clinical-prognostic indicators rather than as a statistically representative characterisation of the metabolic response to capture in this population. These limitations are inherent to the logistical constraints of long-term field capture programmes conducted under operational conditions, and should be addressed in future studies with larger sample sizes and expanded monitoring protocols.

### 4.4. Physical Examination Findings and Capture Safety

Physical injuries associated with restraining trap systems have been systematically categorised by Proulx et al. (2022) [18], who elaborated an Injury Score System based on International Mammal Trapping Standards, distinguishing direct injuries caused by the mechanical action of the snare from indirect injuries resulting from the animal’s behavioural response to capture. The injury profile observed in this cohort is consistent with the lowest severity categories of this system. Using the modified foot injury scoring system of Kuehn et al. (1986) [29], 61.5% of wolves (8/13) presented no excoriations (Grade 0), and the remaining 38.5% showed only superficial lesions (Grade 1 and Grade 2), without skeletal or tendinous involvement. These findings contrast markedly with early baseline data on steel foothold traps, in which Van Ballenberghe (1984) [53] documented severe foot and leg injuries (Class III–IV) in 41% of adult captures, including bone fractures and near-amputation of distal limbs—injury categories entirely absent in the present cohort. These results confirm that direct injuries are minimal when: (i) the snare incorporates springs and swivels that absorb tension and allow rotational movement; (ii) the trap is positioned at sites whose topographic and vegetational characteristics do not predispose to traumatic impact; and (iii) the capture team reaches the trap site within the protocol-defined response time, substantially reducing both direct and indirect injuries [17,18]. The Fremont™ snare, operating within the framework of European humane trapping standards, represents a pragmatic and operationally feasible capture method where its use is legally permissible. Its social acceptability in the European regulatory and public context is further supported by its compliance with Council Regulation (EEC) No. 3254/91, which prohibits leghold traps and establishes humane trapping standards; in this framework, Gethöffer et al. (2022) [54] have also documented the feasibility of net box traps as an alternative capture method. Oral mucosal lesions, observed in 46.2% of individuals, reflect purposeful snare-directed behaviour rather than panic-driven trauma, and are a function of the duration of conscious restraint prior to drug effect. While not negligible, these lesions were superficial and self-limiting in all cases; steps to minimise snare-biting time—principally through faster induction achieved by optimising the approach and drug delivery sequence—may nonetheless be warranted to further reduce this finding in future captures. This proportion is consistent with the 46% rate of tooth, lip, and gum injuries reported by Van Ballenberghe (1984) [53] in wolves captured with steel foothold traps, suggesting that oral mucosal involvement represents an intrinsic feature of mechanical conscious restraint across device types. Preliminary GPS observations in three individuals (M2, F2, F3) documented a transient reduction in movement activity in the immediate post-release period, as reported in Section 3.9. The potential significance of this pattern may be appreciated in the context of baseline movement data available for wolves in the central Apennines. Under normal ranging conditions, resident wolves in this region travel an estimated mean of approximately 27 km per night at average speeds of 2.5 km/h [55], within annual home ranges averaging 104 ± 24 km^2^ for pack members [56]. More directly comparable baseline data are available from 24 h continuous GPS monitoring sessions conducted on individuals belonging to the same Maiella population, yielding mean daily travel distances of 10.8 km (*n* = 6 sessions) for individual F1, 13.3 km (*n* = 3 sessions) for individual F2, and 5.5 km (*n* = 4 sessions) for individual M1 [57]. In the same population, Cavazza et al. (submitted) [58] excluded the first 10 days of post-capture GPS data from home range analyses, explicitly acknowledging post-capture behavioural disruption as a systematic source of movement pattern alteration requiring methodological correction. The post-release displacements of 600–4400 m recorded in the first 48 h for M2, F2, and F3 appear markedly reduced relative to these baseline values, suggesting a transient suppression of normal ranging activity. Given the very limited sample size (*n* = 3), however, no statistical inference can be drawn and these observations should be regarded exclusively as preliminary descriptive data warranting systematic investigation in future studies with adequate sample sizes. A dedicated analysis of post-release movement data for the full cohort will be presented in a separate publication.

## 5. Conclusions

This study provides the first systematic evaluation of a combined Fremont™ humane foot snare and medetomidine-ketamine-acepromazine protocol in free-ranging wolves, reported here for the Apennine wolf (*Canis lupus italicus*). The physiological and hematobiochemical data collected from 13 individuals under operational field conditions document a safe and repeatable immobilization protocol, with no capture-related mortality and no lasting clinical sequelae across the cohort. The findings support the safety and operational feasibility of this combined approach for the Apennine wolf, and provide a baseline physiological and hematobiochemical reference dataset for *C. l. italicus* that may inform future capture protocols and conservation management programmes in Italy and across the European range of the species. Key parameters to monitor during immobilization with this protocol include body temperature—given the individual risk of hyperthermia under alpha-2-ketamine combinations and hypothermia in small juveniles under adverse environmental conditions—heart rate, respiratory rate, and peripheral oxygen saturation, with supplemental oxygen available for individuals presenting SpO_2_ below acceptable thresholds. Venous blood gas analysis, including pH, lactate, pCO_2_, HCO_3_^−^, and base excess, is recommended where logistically feasible, with particular attention to individuals presenting prolonged pre-induction intervals, advanced pregnancy, or signs of physical exertion prior to chemical immobilization. The absence of arterial blood gas capability and blood pressure monitoring represents the primary monitoring gap to be addressed in future capture protocols. Live capture remains indispensable for establishing the scientific and operational framework required to address the increasingly complex challenges of wolf-human coexistence, including conservation monitoring, population health assessment, and individual-based management interventions. Non-invasive methods—including genetic sampling from faecal material, camera trapping, and acoustic monitoring—should be prioritised wherever research objectives can be achieved without physical capture, and live capture reserved for questions that cannot be answered by other means. This work documents a capture and immobilization protocol that, on the basis of current evidence, represents an appropriate and safe approach for the Apennine wolf under the conditions described. The systematic analysis of all available parameters presented here opens both a necessity and an opportunity: to refine field monitoring systems and patient support protocols with the aim of minimising the already low—but non-negligible—probability of capture-related adverse events in future scientific and conservation programmes.

## Figures and Tables

**Table 1:** Captured wolves between June 2010 and July 2017 in Maiella National Park with Fremont and MKA protocol.

Wolf Code MNP	Age at Capture	Date of Capture	Weight (Kg)	Min. Post-Capture Survival Per.
F1	25 months	18 June 2010	28	18 months
F2	18 months	2 December 2010	25	48 months
M1	8 months (*juv*.)	17 January 2011	13.5	8 months
F3	7 months (*juv*.)	11 November 2011	17	7 months
M2	24 months	5 May 2012	38.3	24 months
M3	18 months	15 November 2012	27	48 months
M4	23 months	6 April 2013	33.1	9 months
F4	48 months	8 April 2013	29.4	11 months
M5	6 months (*juv*.)	11 October 2013	19.3	4 months
F5	18 months	23 November 2014	31	26 months
M6	12 months	4 May 2016	36.4	15 months
F6	25 months	3 June 2017	25	57 months
F7	14 months	11 July 2017	22	17 months

**Table 2:** Mean ± SD doses (mg/kg) of medetomidine, ketamine, and acepromazine administered to adult and juvenile Apennine wolves immobilized with the MKA protocol.

	Medetomidine (mg/kg)	Ketamine (mg/kg)	Acepromazine (mg/kg)
Adults (*n* = 10)	0.052 ± 0.005	4.400 ± 0.474	0.142 ± 0.037
Juveniles (*n* = 3)	0.059 ± 0.016	3.989 ± 0.824	0.149 ± 0.040

**Table 3:** Hematological parameters (mean ± SD, min and max) recorded in free-ranging Apennine wolves (*n* = 12/13) immobilized with the MKA protocol.

Parameter	Mean	SD	Min	Max
RBC (×10^12^/L)	7.0	1.1	5.5	8.9
Hemoglobin (g/L)	175.1	26.2	122.0	217.0
Hematocrit (%)	47.8	9.0	33.3	68.2
MCV (fl)	68.8	5.7	59.9	77.6
MCH (pg)	25.1	3.3	22.0	31.3
MCHC (g/L)	370.1	69.9	282.0	492.0
RDW (%)	20.0	8.7	12.7	35.3
HDW (g/L)	300.4	103.0	159.0	550.0
Platelets (×10^9^/L)	244.5	111.2	131.0	526.0
MPV (fl)	9.8	3.0	3.7	14.7
WBC (×10^9^/L)	16.0	5.4	8.2	23.3
Neutrophils (%)	73.2	12.2	45.9	85.8
Lymphocytes (%)	14.6	7.5	7.5	26.8
Monocytes (%)	6.9	6.6	3.6	27.4
Eosinophils (%)	6.4	3.8	0.8	12.3
Basophils (%)	0.8	0.6	0.1	2.5
LUC (%)	0.2	0.2	0.0	0.8

RBC: red blood cell count; MCV: mean corpuscular volume; MCH: mean corpuscular hemoglobin; MCHC: mean corpuscular hemoglobin concentration; RDW: red cell distribution width; HDW: hemoglobin distribution width; MPV: mean platelet volume; WBC: white blood cell count; LUC: large unstained cells.

**Table 4:** Serum biochemistry parameters (mean ± SD, min and max) recorded in free-ranging Apennine wolves (*n* = 12/13) immobilized with the MKA protocol.

Parameter	Mean	SD	Min	Max
Total Protein (g/L)	68	10	59	90
Albumin (g/L)	31	6	16	38
Globulins (g/L)	47	14	32	74
Glucose (mmol/L)	5.27	2.0	2.0	7.0
ALT (IU/L)	79	56	26	195
ALP (IU/L)	92	49	49	195
AST (IU/L)	161	177	52	699
Creatinine (µmol/L)	79.23	28.0	42.0	133.0
BUN (mmol/L)	12.48	6.0	4.0	23.0
Amylase (IU/L)	202	51	133	340
Total Bilirubin (µmol/L)	3.70	1.0	2.0	5.0
Cholesterol (mmol/L)	4.00	1.0	3.0	5.0
Triglycerides (mmol/L)	0.48	0.3	0.0	1.0
Uric Acid (µmol/L)	70.76	14.0	54.0	101.0
Calcium (mmol/L)	2.26	0.3	2.0	3.0
Phosphorus (mmol/L)	1.38	1.0	1.0	3.0
Sodium (mmol/L)	144	11	125	157
Potassium (mmol/L)	4.18	1.0	3.0	5.0

ALT: alanine aminotransferase; ALP: alkaline phosphatase; AST: aspartate aminotransferase; BUN: blood urea nitrogen.

## Data Availability

The data supporting the results reported in this study are not publicly archived but are available upon reasonable request from the corresponding author. All raw data are stored at the Wildlife Research Center, Majella National Park, Via del Vivaio, 65023 Caramanico Terme (PE), Italy, and may be requested by contacting simone.angelucci@parcomajella.it.

## Supplementary Materials

The following supporting information can be downloaded at: https://www.mdpi.com/article/10.3390/ani16111735/s1. S1—Figure S1: An individual receiving intramuscular dart injection during Fremont™/MKA immobilization in Maiella National Park. S2—Video S2: Camera trap footage showing behavioural responses of a restrained wolf (individual M3) during snare restraint prior to capture team arrival. S3—Figure S3: Individual M5 presenting Grade 1 foot lesion (superficial abrasion, total extent < 1 cm) following Fremont™ snare restraint. S4—Figure S4: Individual M4 presenting Grade 2 foot lesion (superficial cut, total extent <2.5 cm) following Fremont™ snare restraint. S5—Video S5: Individual F4 at capture, showing clinical signs of advanced pregnancy including abdominal distension and increased respiratory effort. S6—Figure S6: Individual M6 during clinical monitoring at the handling site. S7—Table S7: Individual physiological parameters (heart rate, respiratory rate, body temperature, SpO_2_) recorded at time points T1, T2, and T3 for each immobilized wolf. S8—Table S8: Complete venous blood gas analysis data (pH, lactate, pCO_2_, HCO_3_^−^, base excess) for the six individuals sampled during immobilization.

## Abbreviations

The following abbreviations are used in this manuscript:

ALP	Alkaline phosphatase
ALT	Alanine aminotransferase (GPT)
AST	Aspartate aminotransferase (GOT)
BE	Base excess
BUN	Blood urea nitrogen
CadVs	Canine adenoviruses
CBC	Complete blood count
CDV	Canine distemper virus
CHV	Canid alphaherpesvirus 1
CK	Creatine kinase
CPV	Canine protoparvovirus 1
GGT	Gamma-glutamyl transferase
GPS	Global Positioning System
GSM	Global System for Mobile Communications
HCO_3_^−^	Bicarbonate
HCT	Haematocrit
HDW	Haemoglobin distribution width
HR	Heart rate
IACUC	Institutional Animal Care and Use Committee
IZSAM	Istituto Zooprofilattico Sperimentale Abruzzo e Molise
ISPRA	Istituto Superiore per la Protezione e la Ricerca Ambientale
IRB	Institutional Review Board
LUCs	Large unstained cells
MAT	Microscopic agglutination test
MCH	Mean corpuscular haemoglobin
MCHC	Mean corpuscular haemoglobin concentration
MCV	Mean corpuscular volume
MK	Medetomidine–ketamine
MKA	Medetomidine-ketamine-acepromazine
MPV	Mean platelet volume
pCO_2_	Partial pressure of carbon dioxide
PaO_2_	Partial pressure of arterial oxygen
PaCO_2_	Partial pressure of arterial carbon dioxide
PLT	Platelet count
PNM	Parco Nazionale della Maiella
RBC	Red blood cell count
RDW	Red cell distribution width
RR	Respiratory rate
SD	Standard deviation
SpO_2_	Peripheral oxygen saturation
T	Rectal temperature
V/Q	Ventilation-perfusion ratio
WBC	White blood cell count
XTZ	Xylazine–tiletamine–zolazepam

## References

[1] Chapron G., Kaczensky P., Linnell J.D.C., von Arx M., Huber D., Andrén H., López-Bao J.V., Adamec M., Álvares F., Anders O. (2014). Recovery of large carnivores in Europe’s modern human-dominated landscapes. Science.

[2] Linnell J.D.C., Cretois B. (2018). The Revival of Wolves and Other Large Predators and Its Impact on Farmers and Their Livelihood in Rural Regions of Europe.

[3] Di Bernardi C., Chapron G., Kaczensky P., Álvares F., Andrén H., Balys V., Blanco J.C., Chiriac S., Ćirović D., Drouet-Hoguet N. (2025). Continuing recovery of wolves in Europe. PLoS Sustain. Transform..

[4] Blanco J.C., Sundseth K. (2023). The Situation of the Wolf (Canis lupus) in the European Union—An In-Depth Analysis.

[5] Zimen E., Boitani L. (1975). Number and distribution of wolves in Italy. Z. Säugetierkunde.

[6] Cimatti M., Ranc N., Benitez-Lopez A., Maiorano L., Boitani L., Cagnacci F., Cengic M., Ciucci P., Huijbregts M.A.J., Krofel M. (2021). Large carnivore expansion in Europe is associated with human population density and land cover changes. Divers. Distrib..

[7] Treves A., Naughton-Treves L., Harper E.K., Mladenoff D.J., Rose R.A., Sickley T.A., Wydeven A.P. (2004). Predicting human-carnivore conflict: A spatial model derived from 25 years of data on wolf predation on livestock. Conserv. Biol..

[8] Apollonio M., Andersen R., Putman R. (2010). European Ungulates and Their Management in the 21st Century.

[9] Mech L.D. (2017). Where can wolves live and how can we live with them?. Biol. Conserv..

[10] Martínez-Abraín A., Jiménez J., Jiménez I., Ferrer X., Llaneza L., Ferrer M., Palomero G., Ballesteros F., Galán P., Oro D. (2020). Ecological consequences of human depopulation of rural areas on wildlife: A unifying perspective. Biol. Conserv..

[11] La Morgia V., Marucco F., Aragno P., Salvatori V., Gervasi V., De Angelis D., Fabbri E., Caniglia R., Velli E., Avanzinelli E. (2022). Stima della Distribuzione e Consistenza del Lupo a Scala Nazionale 2020/2021.

[12] Gervasi V., Aragno P., Salvatori V., Marucco F., Boitani L., Ciucci P. (2024). Estimating distribution and abundance of wide-ranging species with integrated spatial models. Ecol. Evol..

[13] Altobello G. (1921). Fauna dell’Abruzzo e del Molise. Mammiferi. IV. I Carnivori (Carnivora).

[14] Pilot M., Greco C., vonHoldt B.M., Jędrzejewska B., Randi E., Jędrzejewski W., Sidorovich V.E., Ostrander E.A., Wayne R.K. (2014). Genome-wide signatures of population bottlenecks and diversifying selection in European wolves. Heredity.

[15] Silva P., Galaverni M., Ortega-Del Vecchyo D., Fan Z., Caniglia R., Fabbri E., Randi E., Wayne R., Godinho R. (2020). Genomic evidence for the old divergence of southern European wolf populations. Proc. R. Soc. B.

[16] Zanni M., Brogi R., Merli E., Apollonio M. (2023). The wolf and the city: Insights on wolves’ conservation in the Anthropocene. Anim. Conserv..

[17] Arnemo J.M., Ahlqvist P., Andersen R., Berntsen F., Ericsson G., Odden J., Brunberg S., Segerström P., Swenson J.E. (2006). Risk of capture-related mortality in large free-ranging mammals: Experiences from Scandinavia. Wildl. Biol..

[18] Proulx G., Allen B., Cattet M., Feldstein P., Iossa G., Meek P., Serfass T., Soulsbury C., Proulx G. (2022). International Mammal Trapping Standards—Mammal Trapping: Wildlife Management, Animal Welfare & International Standards; Part III: Restraining Trap Systems.

[19] Gese E.M., Terletzky P., Erb J., Fuller K., Grabarkewitz J., Hart J., Humpal C., Sampson B., Young J. (2019). Injury scores and spatial responses of wolves following capture: Cable restraints versus foothold traps. Wildl. Soc. Bull..

[20] Kreeger T.J., Arnemo J.M., Caulkett N.A., Hampton J.O., Meyer L.C.R. (2023). Handbook of Wildlife Chemical Immobilization.

[21] Holz P., Holz R.M., Barnett J.E.F. (1994). Effects of atropine on medetomidine-ketamine immobilization in the gray wolf (*Canis lupus*). J. Zoo Wildl. Med..

[22] Sladky K.K., Kelly B.T., Loomis M.R., Stoskopf M.K., Horne W.A. (2000). Cardiorespiratory effects of four alpha2-adrenoceptor agonist-ketamine combinations in captive red wolves. J. Am. Vet. Med. Assoc..

[23] Angelucci S., Antonucci A., Di Tana F., Innocenti M., Di Domenico G., Madonna L., Smoglica C., Di Francesco C.E., López-Olvera J.R. (2023). Welfare and clinical assessment on physical captures followed by anesthesia in Apennine chamois (*Rupicapra pyrenaica ornata*). Animals.

[24] Di Francesco C.E., Gentile L., Di Pirro V., Ladiana L., Tagliabue S., Marsilio F. (2015). Serologic evidence for selected infectious diseases in Marsican brown bears (*Ursus arctos marsicanus*) in Italy (2004–09). J. Wildl. Dis..

[25] Ebani V.V. (2019). Serological survey of *Ehrlichia canis* and *Anaplasma phagocytophilum* in dogs from central Italy: An update (2013–2017). Pathogens.

[26] Gipson P.S., Ballard W.B., Nowak R.M., Mech L.D. (2000). Accuracy and precision of estimating age of gray wolves by tooth wear. J. Wildl. Manag..

[27] Mech L.D., Boitani L. (2003). Wolves: Behavior, Ecology, and Conservation.

[28] Profico C., Trentini R., Bernabò N., Govoni N., Brugnola L., Sticco M., Iannino F.M., Mattioli M., Lucidi P. (2010). A non-invasive, improved RIA and overt observation in the study of singleton Apennines’ wolf (*Canis lupus*) reproductive behaviour. Anim. Biol. Anim. Husb..

[29] Kuehn D.W., Fuller T.K., Mech L.D., Paul W.J., Fritts S.H., Berg W.E. (1986). Trap-related injuries to gray wolves in Minnesota. J. Wildl. Manag..

[30] Bachmann K., Kutter A., Schefer R., Sigrist N. (2018). Determination of reference intervals and comparison of venous blood gas parameters using a standard and nonstandard collection method in 51 dogs. Schweiz. Arch. Tierheilkd..

[31] Thorneloe C., Bédard C., Boysen S. (2007). Evaluation of a hand-held lactate analyzer in dogs. Can. Vet. J..

[32] Hughes D., Rozanski E.R., Shofer F.S., Laster L.L., Drobatz K.J. (1999). Effect of sampling site, repeated sampling, pH, and PCO2 on plasma lactate concentration in healthy dogs. Am. J. Vet. Res..

[33] Meyer L., Hetem R., Fick L., Matthee A., Mitchell D., Fuller A. (2008). Thermal, cardiorespiratory and cortisol responses of impala (*Aepyceros melampus*) to chemical immobilisation with 4 different drug combinations. J. S. Afr. Vet. Assoc..

[34] Drew M.L., Struthers J.L. (2025). Elevated body temperature associated with ketamine combinations during capture of gray wolves (*Canis lupus*) in Idaho, USA. J. Wildl. Dis..

[35] Morelli J., Briganti A., Fuchs B., Huber D., Evans A.L., Reljić S., Arnemo J.M. (2020). Comparison of two non-invasive arterial blood pressure monitoring techniques in brown bears (*Ursus arctos*). Vet. Anim. Sci..

[36] Talukdar A., Raina P. (2023). Chemical immobilisation of free ranging Tibetan wolf *Canis lupus chanco* with ketamine-xylazine combination in Ladakh, India. J. Threat. Taxa.

[37] Gutema T.M., Atickem A., Lemma A., Bekele A., Sillero-Zubiri C., Zinner D., Farstad W.K., Arnemo J.M., Stenseth N.C. (2018). Capture and immobilization of African wolves (*Canis lupaster*) in the Ethiopian Highlands. J. Wildl. Dis..

[38] Butler M.A., Ballard W.B., Whitlaw H.A. (2006). Physical characteristics, hematology, and serum chemistry of free-ranging gray wolves, *Canis lupus*, in southcentral Alaska. Can. Field-Nat..

[39] Constable P., Hinchcliff K., Demma N., Callahan M., Dale B., Fox K., Adams L., Wack R., Kramer L. (1998). Serum biochemistry of captive and free-ranging gray wolves (*Canis lupus*). J. Zoo Wildl. Med..

[40] Thoresen S.I., Arnemo J.M., Liberg O. (2009). Hematology and serum clinical chemistry reference intervals for free-ranging Scandinavian gray wolves (*Canis lupus*). Vet. Clin. Pathol..

[41] Santos N., Rio Maior H., Nakamura M., Álvares F., Barroso I., Petrucci-Fonseca F., Palacios V., Llaneza L., López-Bao J.V., Godinho R. (2015). Hematology and serum biochemistry values of free-ranging Iberian wolves (*Canis lupus*) trapped by leg-hold snares. Eur. J. Wildl. Res..

[42] Davis A.K., Maney D.L., Maerz J.C. (2018). The use of glucocorticoid hormones or leucocyte profiles to measure stress in vertebrates: What’s the difference?. Methods Ecol. Evol..

[43] Ekstrand C., Pettersson H., Gehring R., Hedeland M., Adolfsson S., Lilliehöök I. (2021). Prednisolone in dogs—Plasma exposure and white blood cell response. Front. Vet. Sci..

[44] Mishler J.M., Emerson P.M., Jameson B., Gunson H.H. (1977). Development of neutrophilia by serially increasing doses of dexamethasone. Br. J. Haematol..

[45] Ottka C., Puurunen J., Lohi H. (2023). Metabolomics during canine pregnancy and lactation. PLoS ONE.

[46] Takeishi M., Uehara M., Yamauchi S., Aoki M., Kanda N., Miyoshi M., Miyoshi T., Iwasaki T., Ohashi F. (2019). Perinatal veterinary medicine-related evaluation in hematological and serum biochemical profiles of experimental beagles throughout pregnancy and parturition. Vet. Med. Sci..

[47] Mattaliano G., Heberlein M., Cruz Benedetti I.C. (2023). Unanticipated hyperkalaemia and associated perioperative complications in three captive grey wolves (*Canis lupus*) undergoing general anaesthesia. Vet. Rec. Case Rep..

[48] Sobrino R., Arnal M.C., Luco D.F., Gortázar C. (2008). Prevalence of antibodies against canine distemper virus and canine parvovirus among foxes and wolves from Spain. Vet. Microbiol..

[49] Almberg E.S., Mech L.D., Smith D.W., Sheldon J.W., Crabtree R.L. (2009). A serological survey of infectious disease in Yellowstone National Park’s canid community. PLoS ONE.

[50] Millán J., López-Bao J.V., García E.J., Oleaga Á., Llaneza L., Palacios V., de la Torre A., Rodríguez A., Dubovi E.J., Esperón F. (2016). Patterns of exposure of Iberian wolves (*Canis lupus*) to canine viruses in human-dominated landscapes. EcoHealth.

[51] Mazzotta E., Bellinati L., Bertasio C., Boniotti M.B., Lucchese L., Ceglie L., Martignago F., Leopardi S., Natale A. (2023). Synanthropic and wild animals as sentinels of zoonotic agents: A study of *Leptospira* genotypes circulating in Northeastern Italy. Int. J. Environ. Res. Public Health.

[52] Žele-Vengušt D., Lindtner-Knific R., Mlakar-Hrženjak N., Jerina K., Vengušt G. (2021). Exposure of free-ranging wild animals to zoonotic *Leptospira interrogans* sensu stricto in Slovenia. Animals.

[53] Van Ballenberghe V. (1984). Injuries to wolves sustained during live-capture. J. Wildl. Manag..

[54] Gethöffer F., Bartels J., Siebert U. (2022). A preliminary assessment of net box traps to catch gray wolves in Lower Saxony, Germany. Wildl. Soc. Bull..

[55] Ciucci P., Boitani L., Francisci F., Andreoli G. (1997). Home range, activity and movements of a wolf pack in central Italy. J. Zool..

[56] Mancinelli S., Boitani L., Ciucci P. (2018). Determinants of home range size and space use patterns in a protected wolf (*Canis lupus*) population in the central Apennines, Italy. Can. J. Zool..

[57] Petrizzelli L. (2011). Status Attuale della Popolazione di Lupo (*Canis lupus*) nel Parco Nazionale della Maiella Attraverso l’Utilizzo Integrato di Diverse Tecniche di Monitoraggio Biologico. Master’s Thesis.

[58] Cavazza S., Antonucci A., Brogi R., Angelucci S., Apollonio M. (2025). Wolf movement patterns across territorial statuses and anthropization gradient: Home range dynamics in a long-established Italian population. Mamm. Biol..

