# Supplementary figures and images for: Physiological Responses and Safety Evaluation of Combined Fremont™ Snare and Medetomidine–Ketamine–Acepromazine Immobilization in Free-Ranging Apennine Wolves (Canis lupus italicus)
Source: Animals (Basel). 2026 Jun 4;16(11):1735. doi: 10.3390/ani16111735 (PMC13255642; doi:10.3390/ani16111735)

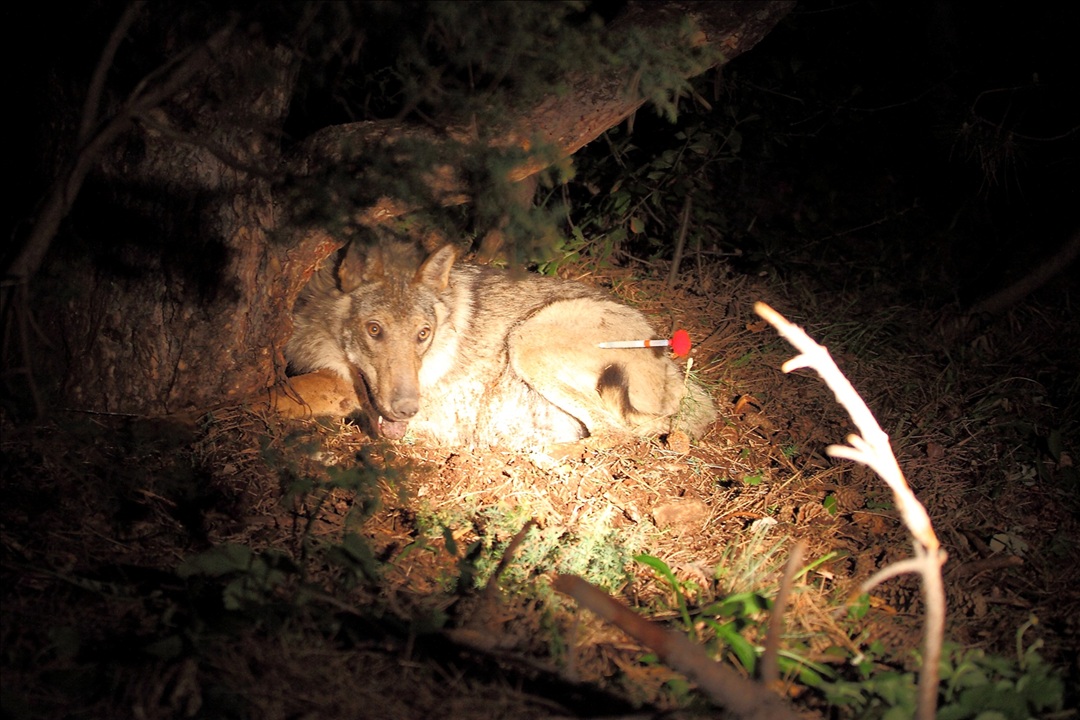

Supplement: Supplementary file 1 [file animals-16-01735-s001.zip › animals-4306293-supplementary-2nd revision/S_1_MNP_wolf_darted.jpg]

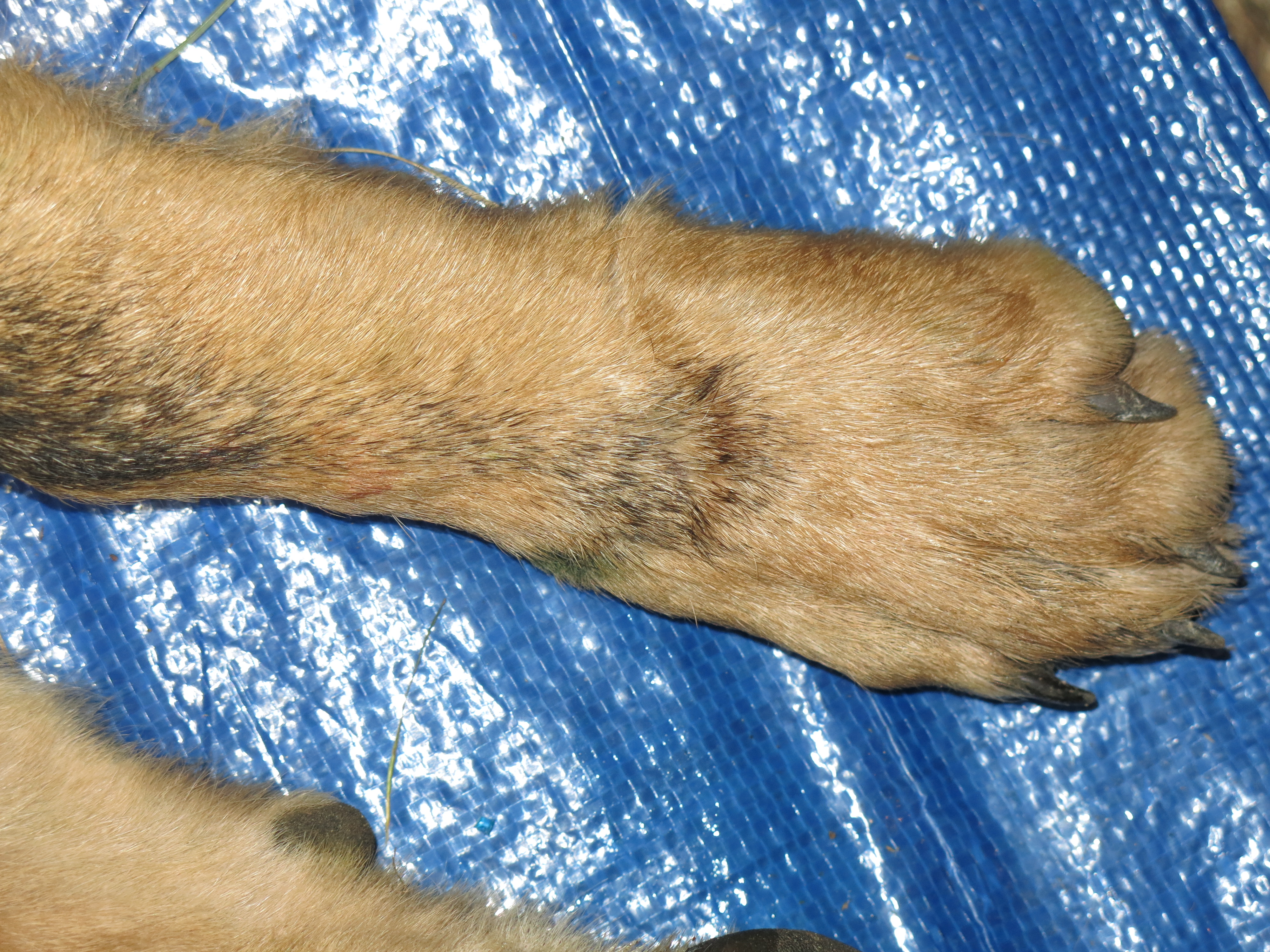

Supplement: Supplementary file 1 [file animals-16-01735-s001.zip › animals-4306293-supplementary-2nd revision/S_3_M5_foot_lesion_1gr.JPG]

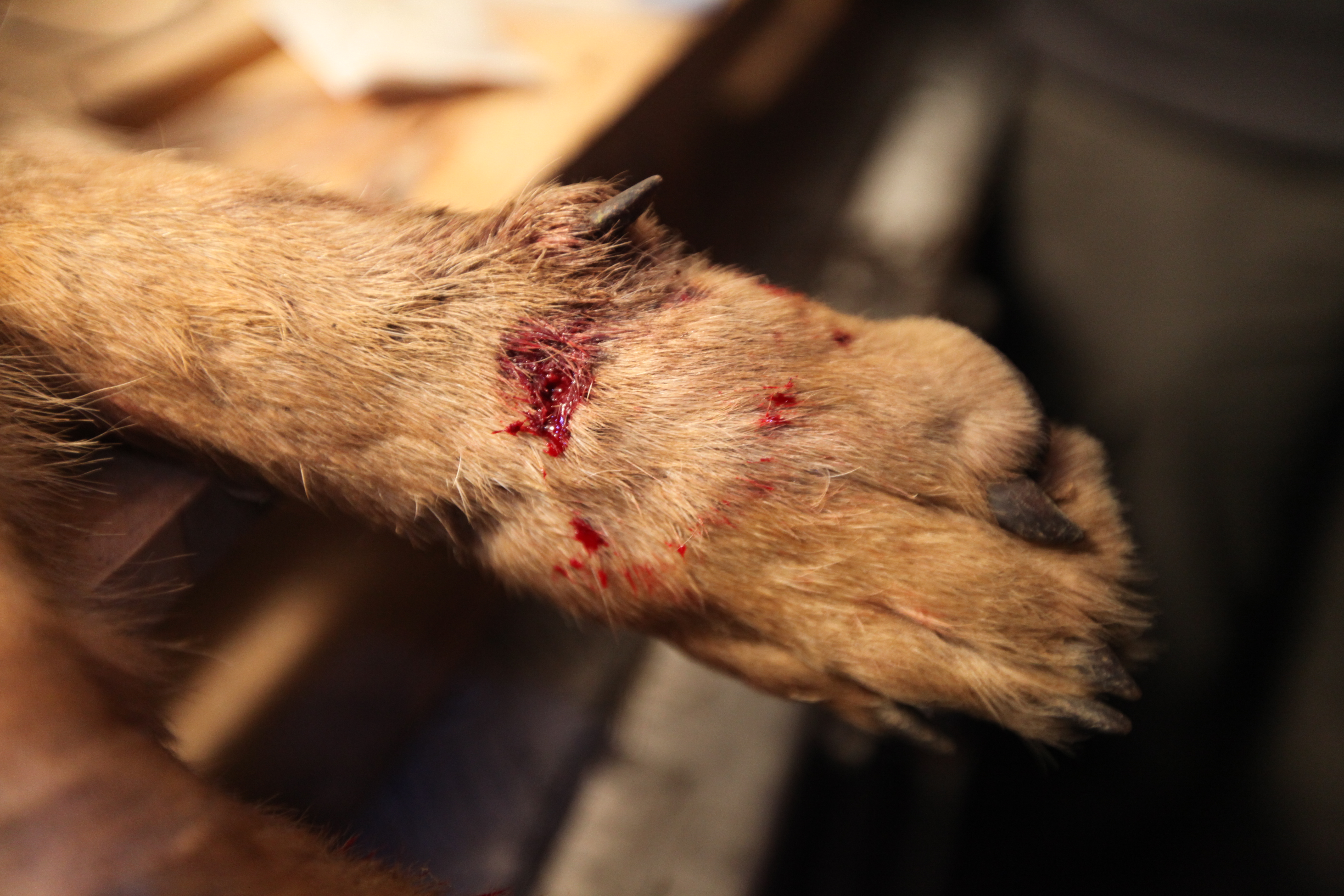

Supplement: Supplementary file 1 [file animals-16-01735-s001.zip › animals-4306293-supplementary-2nd revision/S_4_M4_foot_lesion_2gr.JPG]

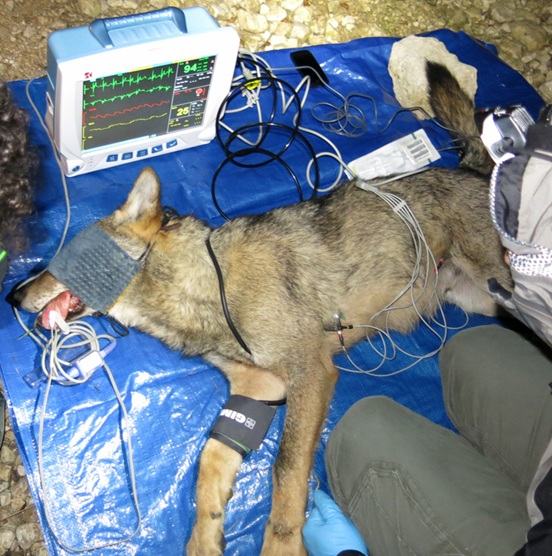

Supplement: Supplementary file 1 [file animals-16-01735-s001.zip › animals-4306293-supplementary-2nd revision/S_6_Clinical_monitoring_M6.jpg]
